# Definition and Characteristics of Mesenchymal Stromal Cells in Preclinical and Clinical Studies: A Scoping Review

**DOI:** 10.1093/stcltm/szab009

**Published:** 2022-02-23

**Authors:** Laurent Renesme, Maria Pierro, Kelly D Cobey, Rhea Mital, Kennedy Nangle, Risa Shorr, Manoj M Lalu, Bernard Thébaud

**Affiliations:** 1 Regenerative Medicine Program, The Ottawa Hospital Research Institute, Ottawa, ON, Canada; 2 Neonatal and Paediatric Intensive Care Unit, M. Bufalini Hospital, AUSL Romagna, Cesena, Italy; 3 Centre for Journalology, Clinical Epidemiology Program, The Ottawa Hospital Research Institute, Ottawa, ON, Canada; 4 School of Epidemiology and Public Health, Faculty of Medicine, University of Ottawa, Ottawa, ON, Canada; 5 Neonatology, Department of Pediatrics, Children’s Hospital of Eastern Ontario (CHEO) and CHEO Research Institute, Ottawa, ON, Canada; 6 Department of Anesthesiology and Pain Medicine, Faculty of Medicine, University of Ottawa, Ottawa, ON, Canada; 7 Department of Cellular and Molecular Medicine, University of Ottawa, Ottawa, ON, Canada

**Keywords:** mesenchymal stromal cell, MSC definition, MSC characteristics, scoping review, preclinical, clinical studies

## Abstract

Mesenchymal stromal cells (MSCs) are widely used in preclinical and clinical research. Despite minimal criteria to define MSCs provided by the International Society for Cell and Gene Therapy (ISCT), concerns have been raised about inconsistent descriptions of cell products used. To address the question “How are MSCs currently defined and characterized?” we conducted a scoping review on original MSC preclinical and clinical studies published over a 3-month period. Selected studies identified from a systematic search of MEDLINE and Embase were categorized as follows: Clinical, Animal, Biology, or Biomaterial studies. Data were extracted from a randomly selected subsample of studies. We extracted information, including epidemiological characteristics of studies, study design, ISCT criteria, and MSC characterization and culture condition. A total of 1053 articles were included and among them, 318 articles were analyzed. Overall, 18% of the articles explicitly referred to the ISCT minimal criteria for MSC. MSC characteristics and culture conditions were inconstantly reported (eg, viability assay reported in only 18% of the articles). Only 20% of documents reported at least 1 functional assay. Clinical studies showed inconsistent completeness in reporting relevant information on the MSC characterization and cell manufacturing processes. These results suggest that further development and implementation of a consensus definition of MSCs and reporting guidelines are needed to enhance rigor, reproducibility, and transparency in MSC research.

## Graphical Abstract

**Graphical Abstract:**
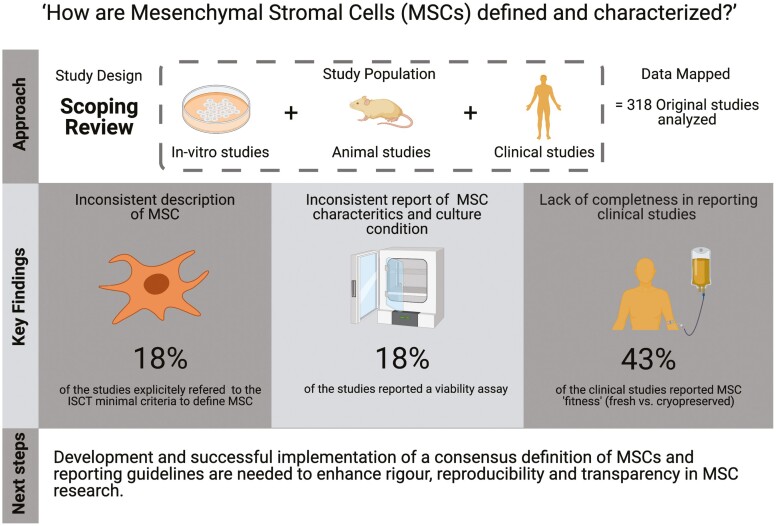

**Significance Statement**

Our scoping review highlights several findings that require the attention of the stem cell community. The minimal criteria to define MSC proposed by the International Society for Cell and Gene Therapy (ISCT) were poorly implemented with inconsistent reporting. More concerning, the clinical studies showed inconsistent completeness in reporting relevant and important information on MSC characterization and cell manufacturing processes. Further efforts are required to ensure the adoption of a consensus definition of MSCs and reporting guidelines in order to enhance rigor, reproducibility, transparency in the MSC literature, and ultimately the safe translation of effective cell-based therapies.

## Introduction

Since mesenchymal stromal cells (MSC) were first tested as a therapeutic agent in 1995,^1^ more than 1300 MSC clinical trials have been registered on clinicaltrials.gov.^2^ Despite highly promising results of MSCs in different preclinical disease models, results of clinical trials using MSCs in various medical conditions have been less encouraging, with currently only 2 clinical approvals for graft versus host disease and Crohn’s associated perianal fistula.^3,4^ Although many issues have contributed to failures in translation (eg, patient characteristics and comorbidities), disparities in MSC characteristics (eg, definition, characterization, immune compatibility, cell viability, and dose) appear to be critical factors.^4^ Within the clinical trials that have tested MSC therapy there has been significant heterogeneity in reporting of products used, despite attempts from the International Society for Cell and Gene Therapy (ISCT) to provide minimal criteria to define MSC.^5,6^ For example, a report from the Food and Drug Administration showed important differences in cell surface marker characterization, product bioactivity assessment, as well as tissue sourcing and product manufacturing.^7^ Compounding the issue of heterogeneous cell products is incomplete reporting.^8-12^

To better address clinical translation, reproducibility, and transparency in the field of MSC research, the scientific community needs to agree a consensus definition of MSCs and supports its dissemination and implementation. The absence of a consensus definition will lead to ongoing difficulties in study quality assessment, comparison between studies, extrapolation from study findings, and even possibly influence the results of preclinical and clinical reports.

Here we seek to address the question “How are MSCs currently defined and characterized?” The objective of the current study is to describe how MSCs are defined and characterized in preclinical and clinical research assessing MSCs’ therapeutic potential. In this scoping review, we used a systematic search to map the current literature and identified key concepts and knowledge gaps.^13,14^ This scoping review is the first step in a larger research program that seeks to establish a new consensus definition of MSCs.^15^ Our results will inform a subsequent Delphi study^16^ to establish and implement an international definition of MSCs.

## Materials and Methods

Our research protocol was drafted according to the methodological framework for scoping reviews proposed by Arksey et al and adapted by Levac et al^13,14^ and further updated by the Joanna Brigg’s Institute.^17^ This protocol was registered prospectively using the Open Science Framework^18^ (see https://osf.io/3dsqx/) The data charting form was continuously updated from the protocol version as part of an iterative process as data was charted. The methods and findings of this study are reported in accordance with the PRISMA Extension for Scoping Review.^19^ Study materials and data can be found here: https://osf.io/3dsqx/.

### Research Question

Our research question was: “How are MSCs currently defined and characterized in published preclinical and clinical studies?” The purpose of this scoping review was to describe how MSCs are described and defined in a sample of preclinical and clinical research literature.

### Search Strategy: Identifying Relevant Studies

We identified relevant original preclinical and clinical MSC studies published over a 3-month period (March 1-May 31, 2020). A 3-month period was chosen a priori due to the large number of publications related to MSCs.

The search strategy (see Supplemental material) was developed by an experienced information specialist (R.S.) and further refined through team discussion. This search strategy was modified from previous systematic reviews of MSCs by our group,^10,20^ and underwent Peer Review of Electronic Search Strategy (PRESS) to ensure adequate sensitivity and specificity.^21^ To identify potentially relevant studies, the following MEDLINE and Embase were searched. We ran simultaneous searches of Ovid Medline All and Embase using a broad search strategy. MeSH terms for MSCs were searched along with synonymous text words in the titles or abstracts such as multipotent stromal cells.

Results of this search were limited to English language articles, de-duplicated, and then uploaded into DistillerSR^22^ (Evidence Partners, Ottawa), a cloud-based, audit-ready software that facilitates screening and selection of articles and allows transparent and reproducible work.

### Study Selection

#### The Study Population

The study population included original preclinical and clinical MSC studies. To be included, articles needed to report original research using MSC as a main intervention/focus and/or assess its therapeutic potential (for Animal and Clinical studies). We excluded studies if MSC were not the main intervention/focus, if the study did not investigate mesenchymal stromal/stem cells and if it was not an original study (eg, editorial, review). Systematic reviews and meta-analyses were also excluded.

#### Screening

Two independent reviewers (L.R., M.P.) performed the study selection using DistillerSR. For each screening step (title and abstract and full text), calibration exercises were done on 10 random articles to ensure inter-reviewer reliability. Conflicts were resolved by consensus among screeners, or if needed by a third independent reviewer (B.T.). First, the 2 independent reviewers screened article titles and abstracts in duplicate using an initial screening questionnaire. Subsequently, full-text screening for all the articles retained was conducted against our eligibility criteria. Selected studies were stratified according to 3 categories: In Vitro, Animal, and Clinical studies. In vitro studies were stratified using the following categories: MSC Biology, MSC, and Biomaterial. Finally, if the number of articles included a category was >100, we used a random sample of 25% for data charting. The random sample was selected using a function in DistillerSR.

### Charting the Data

#### Data-Extraction Forms

Three data-extraction forms were developed *a priori* for each category (Clinical, preclinical Animal, and preclinical In Vitro) and pilot-tested by our team. These forms were designed to capture epidemiological characteristics of the original study and detailed information on MSC descriptions and their use. The data-extraction forms collected information on:

- *Epidemiological characteristics.* Information on publication year, corresponding author (name, email, and country of affiliation), and funding (reported funding and funding source) were captured.
- *Study design (Clinical and Animal categories only).* Information on the disease studied, intervention group (MSC dose, administration route, concentration, etc.), and control group were collected.
- *MSC description and reference to ISCT minimal criteria.* This section of the form was designed to evaluate how authors referred to the ISCT criteria (eg, plastic adherence, cell markers, tissue source, differentiation assays) and provide information on which criteria were used and how detailed were these criteria.
- *MSC characteristics and culture condition.* This section was designed to inform the MSC characteristics (species sources, compatibility for Clinical and Animal studies, “fitness,” and viability assessment) and what were the culture condition, including a number of passages prior to MSC administration, cell confluence during culture, oxygen condition, medium, and serum used.

The full data-extraction forms are accessible on the Open Science Framework (https://osf.io/3dsqx/).

#### Data Charting Process

Two independent reviewers (L.R., M.P.) performed the data extraction using a single charting and audit approach using the quality control function in DistillerSR. Each reviewer charted half of the articles and audited the other half. In case of disagreement between the reviewers, a third independent reviewer (B.T.) was consulted. The extraction forms were piloted on 5 random studies of each sample (in vitro, animal, and human) to ensure the approach to data charting was consistent and in line with the research question and purpose. Then a calibration exercise was done on the next 10 articles for each category. The team discussed results, and the data charting form was continuously updated in an iterative process in order to be inclusive of other aspects of the cell characterization, manufacturing, delivery, etc. not listed a priori.

Extracted information will be used to generate our initial set of items for the future Delphi survey. In addition, the corresponding author’s names/email and affiliation country were extracted from all original articles selected for charting. These authors will be contacted by a member of the research team to participate in the Delphi survey.

### Collating, Summarizing, and Reporting Results

We conducted our data analysis distinctly for each study design group (Biology, Biomaterial, Animal, and Clinical studies) and involved both quantitative (ie, frequencies) and qualitative (ie, thematic analysis) methods. We have reported frequencies and percentages for original studies’ epidemiological characteristics (country listed in the first stated affiliation of the first listed corresponding author on each article for each study category, funding sources presented, and disease model) and study design (intervention group with MSC route of administration and dose, control group, cell nomenclature). Study participants are described as species for preclinical Animal studies (frequency and percentages) or participants’ age category for Clinical studies (pediatric, adult, or both). MSC description, frequencies, and percentages were reported for: ISCT minimal criteria for MSC (and functional matrix assays for Clinical studies), and MSC characteristics (tissue source, “fitness,” and culture condition).

At the request of reviewers, we also conducted an unplanned post hoc analysis to provide a description of how completeness of reporting related to journal impact factor. To do so, we obtained journal impact factors for included journals from Clarivate’s Journal Citation Reports for the year 2020 (https://jcr.clarivate.com). An additional post hoc analysis was done to compare the completeness of reporting between randomized controlled trials (RCT) and other clinical studies.

## Results

### Study Selection

**Figure 1.**
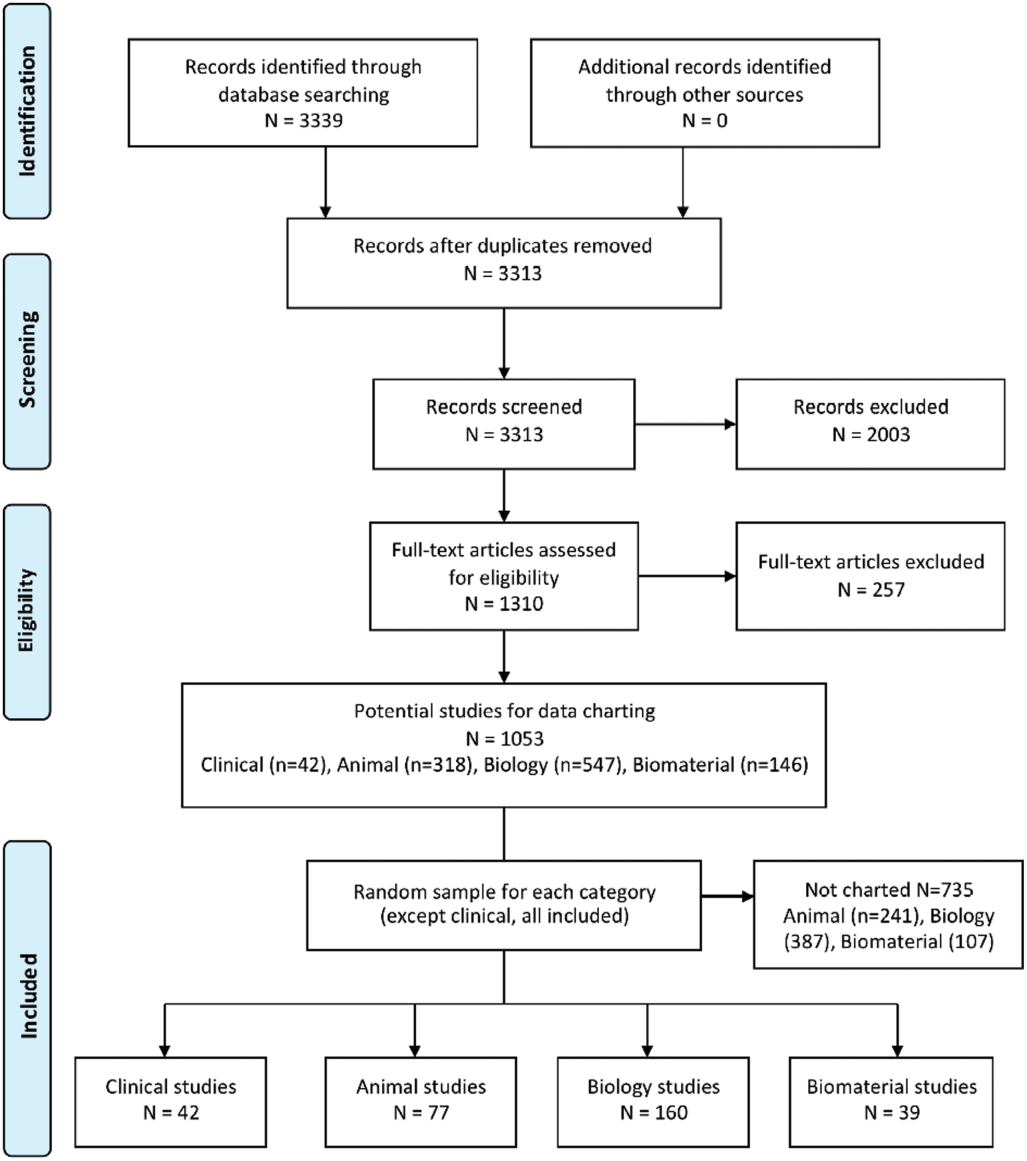
Preferred Reporting Items for Systematic Reviews and Meta-Analyses (PRISMA) flow diagram.

In total, 3339 potential articles were identified and following screening 1053 articles met our inclusion criteria (Fig. 1). Among these articles, we selected all the included Clinical studies, and random samples of the Animal, Biology, and Biomaterial studies to conduct data extraction. A total of 318 articles were included for charting: 42 Clinical studies, 77 Animal studies, 160 Biology studies, and 39 MSC and Biomaterial studies. For data charting, the weighted overall Kappa was 0.93 for Clinical studies, 0.98 for Animal studies, 0.95 for Biology studies, and 0.89 for Biomaterial studies.

### Epidemiological Characteristics

Among all the selected articles (*n* = 318), the top 3 countries were China (37%), US (13%), and Korea (6%). Funding was reported in 92% of the articles, 39% of the articles reported multiple funding sources, and Government was the most frequent source of funding (reported in 64% of the articles). A summary of article epidemiological characteristics is presented in Table 1 and more details are provided in Supplemental Table S1.

**Table 1.** Article epidemiological characteristics.

	Clinical(*n* = 42)	Animal (*n* = 77)	Biology *n* = 160)	Biomaterial (*n* = 39)	All articles (*n* = 318)
Top 3 journals (*n*)	- Stem Cell Research & Therapy (3)- Stem Cells Translational Medicine (2)- Knee Surgery, Sports Traumatology, Arthroscopy (2)	- Stem Cell Research & Therapy (3)- International Journal of Stem Cells (3)- Stem Cells and Development (3)- Stem Cells International (3)	- Stem Cell Research & Therapy (6)- Cells (5)- Molecular Medicine Reports (4)	- Journal of Materials Chemistry B (11)- ACS Biomaterials Science & Engineering (3)- ACS Applied Materials & Interfaces (3)	- Stem Cell Research & Therapy (13)- Journal of Materials Chemistry B (12)- Stem Cells International (8)
Top 3 countries (*n*)	- China (7)- US (6)- Spain (4)	- China (39)- US (6)- India (4)	- China (58)- US (19)- Germany (13)- Korea (13)	- China (13)- US (6)- Iran (3)	- China (117)- US (42)- Korea (20)
Funding reported	32 (76)	77 (100)	145 (91)	37 (95)	291 (92)
Multiple sources	11 (34)	32 (42)	60 (41)	20 (54)	123 (42)
Funding sources					
Government	15 (36)	53 (69)	107 (67)	28 (72)	203 (64)
Academic	7 (17)	26 (34)	50 (31)	21 (54)	104 (33)
Foundation	9 (21)	9 (12)	31 (19)	6 (15)	55 (17)
Industry	3 (7)	3 (4)	5 (3)	2 (5)	13 (4)
Hospital	9 (21)	9 (12)	8 (5)	1 (3)	27 (8)
Reported as not funded	6 (14)	2 (3)	5 (3)	0 (0)	13 (4)

Funding data presented as *n* (%).

### General Study Characteristics

#### Clinical Studies

Forty-two clinical studies were analyzed. Thirty-two (76%) were observational studies (27 descriptive studies and 5 analytical studies—1 case-control and 4 cohort studies). There were 10 (24%) experimental studies, of which 9 were RCT.

Of the 42 studies, 35 (83%) studies used an adult population, 2 (5%) a pediatric population, and 5 (12%) both adult and pediatric populations. Regarding the disease model, the 3 most frequently investigated systems were musculoskeletal system (29%), respiratory system (17%), and nervous system and genitourinary systems, both reported in 14% of the studies (Supplemental Table S2). These 42 Clinical articles described an important variety of administration protocols, with wide ranges for MSC doses and the number of doses administered (Table 2). MSC dose was reported in cells/kg (39%) with a median dose of 10^6^ cells/kg (range 10^5^ to 10^7^ cells/kg) or in total cells per dose (58%) with a median dose of 2.5 × 10^7^ cells per dose (range 5 × 10^5^ to 325 × 10^6^ cells per dose). Multiple MSC doses were used in 29% of the Clinical articles with a median of 3.5 doses (range 2-25 doses). A control group was reported in 15 studies (36%). Control groups included a vehicle placebo (5 studies, 33%), standard care with no placebo (8 studies, 53%), other cell types (1 study, 7%), or unreported (1 study, 7%).

**Table 2.** Intervention group in clinical studies.

	*N* (%)
Administration route reported	41 (98)
Multiple administration routes	4 (10)
Intravenous	13 (32)
Infusion rate reported in 6 of 13 studies (46%) using IV route	
Intratracheal	1 (2)
Intramuscular	4 (10)
Intrathecal	3 (7)
Intra-articular	3 (7)
Other routes	21 (51)
Cardiovascular system (intra-myocardium)	2 (10)
Digestive system	3 (14)
Ear-nose-throat	1 (5)
Eye	1 (5)
Genitourinary system	2 (10)
Musculoskeletal tissue (tendon)	8 (38)
Nervous system	2 (10)
Skin and subcutaneous tissue	2 (10)
MSC dose reported	38 (90)
Dose in cells/kg	15 (39)
Range 10^5^ to 10^7^ cells/kg	
Dose in a total amount of cell administered	22 (58)
Range 5 × 10^5^ to 325 × 10^6^ cells per dose	
Other dose reported	1 (3)
Dose reported in µL/cm^2^ of the defect area	
Different dose groups used in the study	15 (39)
Single administration	28 (74)
Multiple administration	11 (29)
Range 2-25 doses	
MSC concentration reported	13 (31)
Use of dimethyl sulfoxide (DMSO) reported	9 (21)

Abbreviations: IV, intra-venous; MSC, mesenchymal stromal cells.

#### Animal Studies

Seventy-seven studies were randomly selected and analyzed. All the articles reported the animal species used for the model; most of them were rodents: rat, *n* = 45 (58%); mouse, *n* = 24 (31%); rabbit, *n* = 5 (6%); other (swine, sheep, and dog), *n* = 3 (4%). Regarding the disease model, the 3 most reported systems were nervous system (25%), musculoskeletal system (16%), and cardiovascular system (12%). The different disease models used in the animal studies are presented in Supplemental Table S2. For the intervention group, the administration route was reported in all studies and 91% (70/77) of the animal studies reported the MSC dose. The most common administration route was intravenous, reported in 43% of the articles. MSC dose was reported in cells/kg in 24% of the articles with a median dose of 2.25 × 10^6^ cells/kg (range 1.25 × 10^5^ to 10^7^ cells/kg) or in total cells per dose in 71% of the articles, with a median dose of 10^6^ cells per dose (range 3 × 10^4^ to 10^7^ cells per dose). Sixteen percent of the articles reported using multiple MSC doses, with a median of 3 doses (range 2-6 doses). The reported intervention group for animal studies is detailed in Supplemental Table S3. A control group was reported in 76 studies (99%) with vehicle injection (67%) or no injection (28%).

### MSC Description and Reference to the ISCT Minimal Criteria

#### Cell Nomenclature

Mesenchymal stem cell was used more frequently than MSC (Clinical 69 vs. 24%; Animal 82 vs. 17%, Biology 73 vs. 24%; Biomaterial 82 vs. 15%, all articles 76 vs. 21%). A small proportion of the studies used both terms (2% of all the articles).

#### Reference to ISCT Criteria and Recommendations

**Figure 2.**
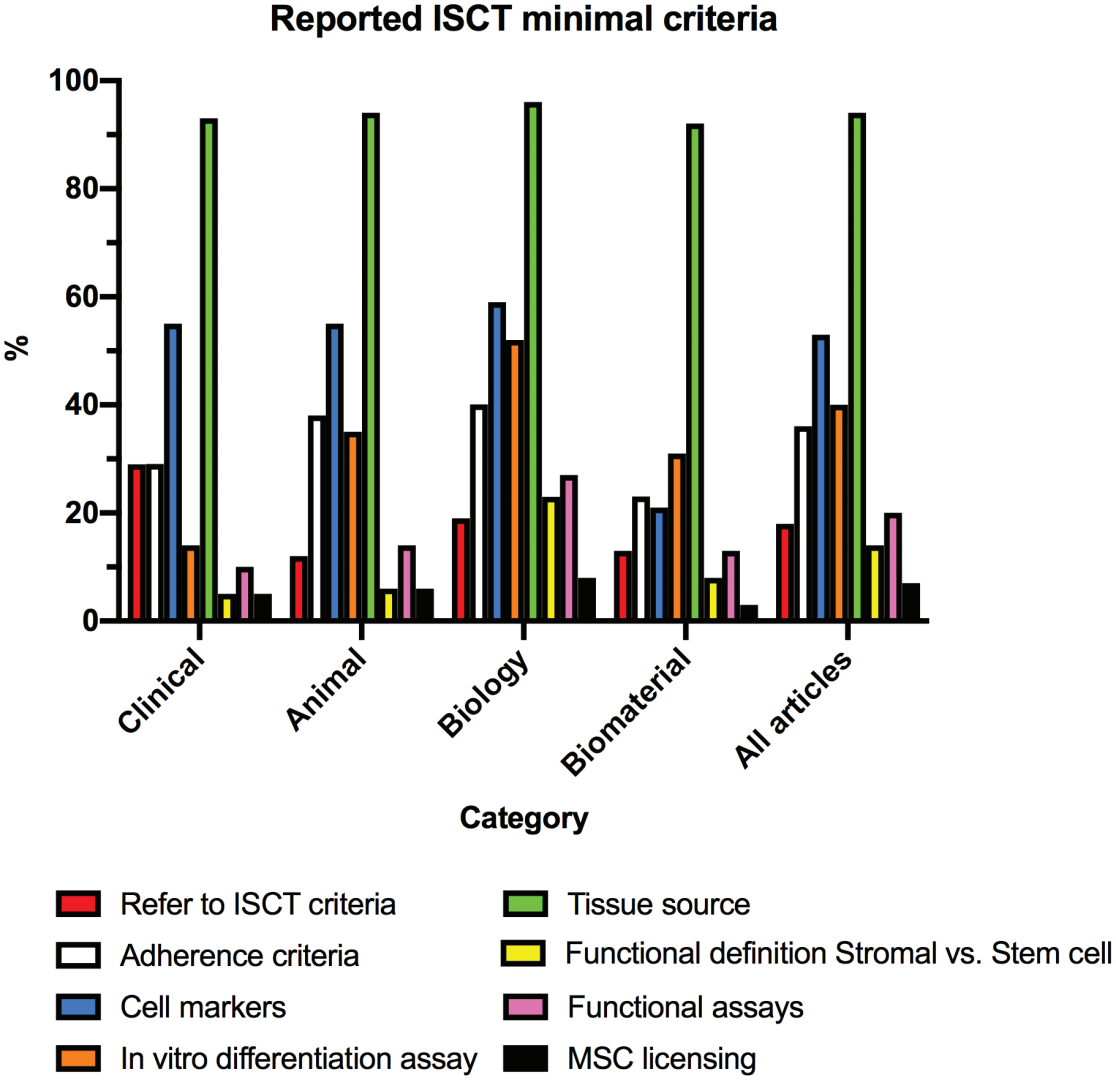
Reported International Society for Cell and Gene Therapy (ISCT) minimal criteria for mesenchymal stromal cells (MSC). This graph shows, for each study category, the percentage of studies reporting the criterion. The criterion “refer to ISCT criteria” refers to the number of studies where the authors stated that they were using the ISCT criteria.

Overall, only 18% of the articles explicitly referred to the ISCT minimal criteria for MSC, with the highest percentage of articles in the Clinical category (29%) and the lowest for the Animal category (12%). The reported ISCT criteria according to each study category are presented in Fig. 2 and detailed in Table 3. To further explore the completeness in reporting the initial minimal criteria to define MSC from the ISCT (plastic adherence, cell markers, and in vitro differentiation assay), we described its association with the journal impact factor in Supplemental Table S4, and comparison between RCT and other clinical studies is presented in Supplemental Table S5. Among all the articles, 55 different positive or negative markers were used to define MSC. The top reported positive and negative cell markers are presented in Supplemental Fig. S1, and Supplemental Table S5 compared the top reported cell markers between RCT and other clinical studies. In addition to the minimal criteria to define MSC, to ensure product consistency, the ISCT also recommends to report tissue source as well as functional assays including in vitro MSC licensing with pro- inflammatory cytokines in order to mimic the in vivo environment in patients with systemic inflammation or abnormal immune response.^23^ Tissue source was reported in 94% of all the articles with a wide variety of tissue used as MSC source. MSC licensing was used in 7% of all the articles, the molecule or substance used was always reported and a resting MSC was used as control in 24% of the articles reporting MSC licensing.

**Table 3.** Reported International Society Cell and Gene Therapy (ISCT) minimal criteria to define mesenchymal stromal cells (MSC).

	Clinical (*n* = 42)	Animal (*n* = 77)	Biology (*n* = 160)	Biomaterial (*n* = 39)	All articles (*n* = 318)
Refer to ISCT criteria	12 (29)	9 (12)	31 (19)	5 (13)	57 (18)
Studies without any criteria	2 (5)	5 (7)	6 (4)	3 (8)	16 (5)
Adherence criteria reported	12 (29)	29 (38)	64 (40)	9 (23)	114 (36)
Plastic	6 (50)	7 (24)	14 (22)	2 (22)	29 (25)
Surface not reported	6 (50)	22 (76)	50 (78)	7 (78)	85 (75)
Cell markers reported	23 (55)	42 (55)	95 (59)	8 (21)	168 (53)
In vitro differentiation assay reported	6 (14)	27 (35)	83 (52)	12 (31)	128 (40)
Adipocyte	6 (100)	24 (89)	60 (72)	8 (67)	98 (77)
Chondrocyte	4 (67)	14 (52)	39 (47)	5 (42)	62 (48)
Osteoblast	6 (100)	24 (89)	77 (93)	10 (83)	117 (91)
Tri-lineage	4 (67)	13 (48)	37 (45)	4 (33)	58 (45)
Tissue source reported	39 (93)	72 (94)	153 (96)	36 (92)	300 (94)
Bone marrow	17 (44)	40 (56)	95 (62)	26 (72)	178 (59)
Adipose tissue	9 (23)	9 (13)	29 (19)	5 (14)	52 (17)
Umbilical cord	10 (26)	18 (25)	17 (11)	2 (6)	47 (16)
Placenta	0 (0)	3 (4)	0 (0)	1 (3)	4 (1)
Amnion	0 (0)	1 (1)	1 (1)	1 (3)	3 (1)
Synovial	0 (0)	2 (3)	4 (3)	0 (0)	6 (2)
Peripheral blood	0 (0)	0 (0)	3 (2)	0 (0)	3 (1)
Other	4 (10)	1 (1)	26 (17)	1 (3)	32 (11)
Functional definition MSC stromal versus stem reported	2 (5)	5 (7)	36 (23)	3 (8)	46 (15)
Self-renewal assay	2 (100)	5 (100)	25 (69)	3 (100)	35 (76)
Multilineage differentiation	0 (0)	0 (0)	20 (56)	0 (0)	20 (44)
Functional assays reported	4 (10)	11 (14)	43 (27)	5 (13)	63 (20)
Quantitative RNA analysis	0 (0)	4 (36)	28 (65)	4 (80)	36 (57)
Mixed lymphocyte reaction	2 (50)	1 (9)	3 (7)	0 (0)	6 (10)
MSC secretome analysis	0 (0)	2 (18)	14 (33)	0 (0)	16 (25)
Migration assay	0 (0)	8 (73)	7 (16)	4 (80)	19 (30)
Other	2 (50)	2 (18)	2 (5)	0 (0)	6 (10)

Data presented as *n* (%).

For in vitro differentiation assay, studies can report none, 1, 2, or tri-lineage. The data are reported in this table as the count number for each lineage differentiation.

Tissue source: some studies reported the use of different tissue sources, the description of the tissue source is shown as count number and therefore can be superior to the number of studies reporting tissue source.

“Other” tissue source details:

Clinical: menstrual blood (2), endometrial tissue (1), gingival connective tissue (1).

Animal: cardiac tissue (1).

Biology: abdominal aortic aneurysm wall (1), amniotic fluid (2), bone fracture site (1), dental apical papilla tissue (1), dental follicle (1), dental pulp (1), dermis (3), endometrial tissue (1), gingival tissue (2), hair follicle (1), peri-cardiac fat (1), liver (1), peri-tumor normal tissue (1), olfactory mucosa (1), Teeth (1), tongue epithelium (1), tonsil (3), femoral marrow fat (1), coronary corium (1), alveolar bone (1).

Biomaterial: dental pulp (1).

### MSC Characteristics and Culture Conditions

**Figure 3.**
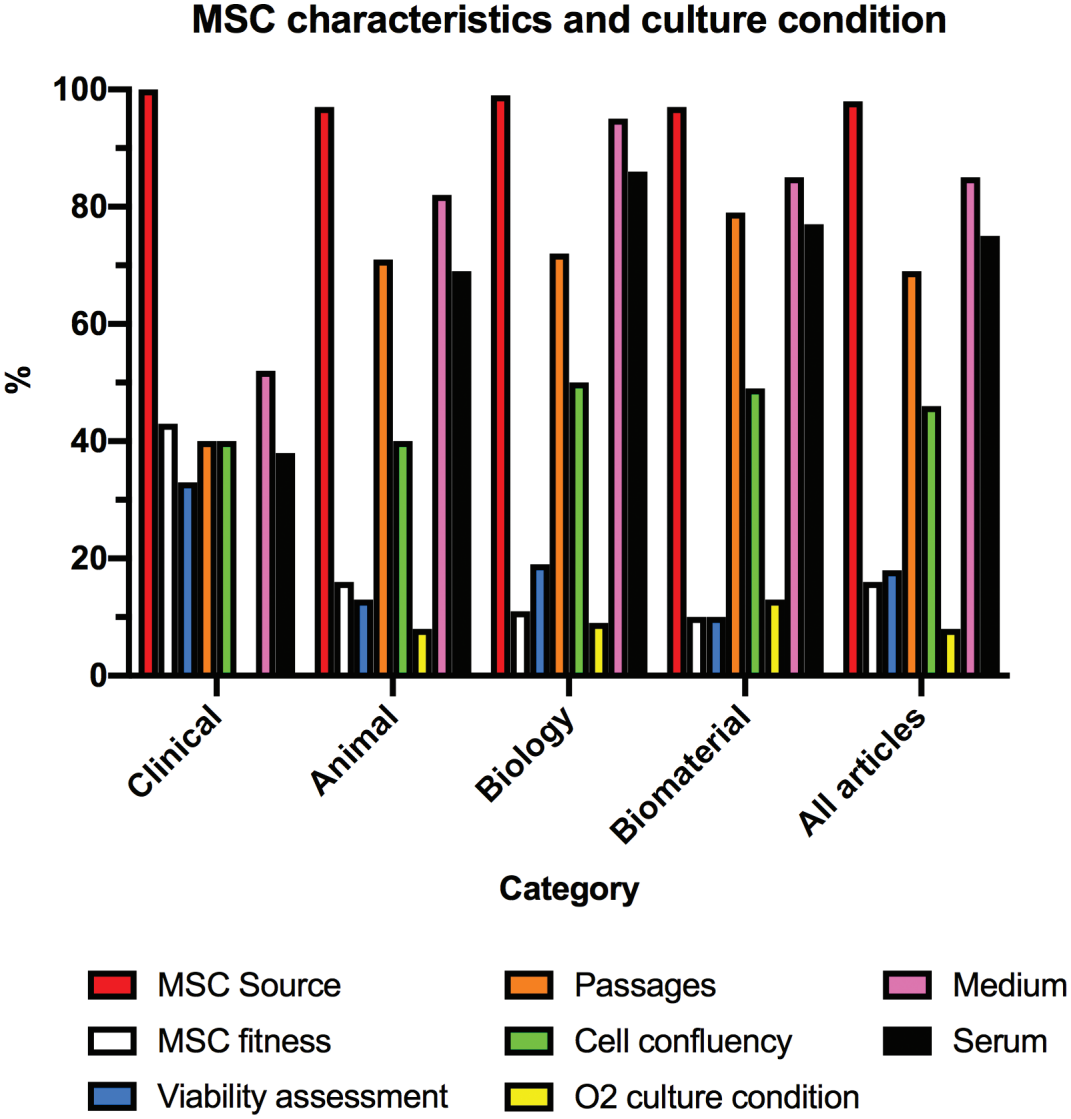
Reported mesenchymal stromal cells (MSC) characteristics and culture condition. For MSC characteristics, the following items were assessed: MSC source (eg, patient, donor, commercial), MSC fitness (fresh or cryopreserved MSC), viability assessment prior to MSC use. For culture condition, the following items were assessed: number of cell passages prior to cell use/administration, cell confluence before cell harvest, oxygen (O_2_) condition for culture (5% vs. 21% of oxygen), type of medium, and serum use.

The reported MSC characteristics and culture conditions are presented in Fig. 3 and detailed in Table 4. For MSC characteristics, MSC source (ie, human, animal or commercial) was almost always reported (in 98% of all articles), but MSC “fitness” (ie, fresh or cryopreserved) as well as MSC viability assessment prior to administration were reported in only 16% and 18% of all articles, respectively. Reporting items describing the culture condition were inconsistently reported, culture medium type being the most reported (in 85% of all articles), and oxygen culture condition being the least reported (8% of all articles).

**Table 4.** Reported mesenchymal stromal cells (MSC) characteristics and culture condition.

	Clinical (*n* = 42)	Animal (*n* = 77)	Biology (*n* = 160)	Biomaterial (*n* = 39)	All articles (*n* = 318)
MSC source reported	42 (100)	75 (97)	158 (99)	38 (97)	313 (98)
Patient/donor	41 (98)	34 (45)	113 (72)	24 (63)	212 (68)
Animal	N/A	37 (49)	51 (32)	13 (34)	101 (32)
Commercial	3 (7)	10 (13)	37 (23)	10 (26)	60 (19)
Compatibility reported	38 (90)	53 (69)	N/A	N/A	91 (76)
Autologous	27 (71)	0 (0)			27 (30)
Matched allogenic	3 (8)	1 (2)			4 (4)
Unmatched allogenic	8 (21)	19 (36)			27 (30)
Xenogenic	N/A	33 (62)			33 (36)
MSC fitness reported	18 (43)	12 (16)	17 (11)	4 (10)	51 (16)
Fresh	4 (22)	0 (0)	3 (18)	0 (0)	7 (14)
Cryopreserved	14 (78)	12 (100)	14 (82)	4 (100)	44 (86)
Viability assessment reported	14 (33)	10 (13)	30 (19)	4 (10)	58 (18)
Number of passages reported	17 (40)	55 (71)	115 (72)	31 (79)	218 (69)
Range (*n* passages)	1-7	1-20	1-38	2-14	1-38
Cell confluence reported	17 (40)	31 (40)	80 (50)	19 (49)	147 (46)
Range (%)	70-90	30-100	50-100	60-90	30-100
O_2_ culture condition reported	0 (0)	6 (8)	14 (9)	5 (13)	25 (8)
O_2_ 5%	0 (0)	2 (33)	4 (29)	0 (0)	6 (24)
O_2_ 21%	0 (0)	4 (67)	10 (71)	5 (100)	19 (76)
Culture medium reported	22 (52)	63 (82)	152 (95)	33 (85)	270 (85)
α-MEM	8 (36)	8 (13)	32 (21)	7 (21)	55 (20)
DMEM	1 (5)	23 (37)	48 (32)	13 (39)	85 (31)
DMEM/F12	7 (32)	9 (14)	25 (16)	2 (6)	43 (16)
LG-DMEM	3 (14)	12 (19)	20 (13)	6 (18)	41 (15)
Serum use reported	16 (38)	53 (69)	138 (86)	30 (77)	237 (75)
No serum	1 (6)	1 (1)	0 (0)	0 (0)	2 (1)
FBS	11 (69)	52 (68)	130 (94)	30 (100)	223 (94)

Data presented as *n* (%).

*MSC fitness and clinical studies.* For cryopreserved MSC, 50% reported frozen/thawed/MSC administration and 50% reported frozen/thawed/cultured/MSC administration.

*MSC sources reported.*

Some studies reported the use of different sources.

Animal sources—Animal studies: rat (19), mouse (7), rabbit (4), dog (1), pig (1), sheep (1), unknown (4); Biology studies: rat (18), mouse (20), bovine (1), dog (3), goat (1), horse (1), ovine fetus (1), rabbit (1), shrew (1); Biomaterial studies: rat (4), rabbit (3), mouse (3), sheep (1).

Commercial, details on product and company names for clinical and animal studies—Clinical studies: Bionet Corp. (1), Cartistem Medipost (1), Basic Medical Sciences (1); Animal studies: Cyagen Biosciences (3), Lonza (1), hUC-MSC CHA Biotech (1), Shanghai Yiyan Biotechnology (1), Shanghai Saibaikang Biotechnology (1), Noor Genetics Laboratory of Ahva (1), Kalang Technology (1), RoosterBio (1).

*Serum in clinical studies.* Four studies reported other serum than FBS: autologous serum, human serum B, HyClone, newborn calf serum.

Abbreviations: DMEM, Dulbecco’s modified Eagle’s medium; LG-DMEM, low-glucose DMEM; FBS, fetal bovine serum; MEM, minimum essential medium; O_2_, oxygen.

## Discussion

The aim of this scoping review was to describe how MSC are currently defined in preclinical (in vitro and animal studies) and clinical studies and to describe the characteristics of this published literature. In our selected articles, we found that only 29% of the clinical studies and 18% of all articles explicitly refer to the ISCT minimal criteria for MSC, and found important variations in criteria used to define MSC and the tissue sources, cell characteristics, and culture conditions. Both clinical and animal studies showed important variation in MSC dose. For example, in clinical studies, we found a two-log difference between the lowest and the highest dose reported and a >600-fold difference in the studies reporting total cells infused. In addition to these variations, we found that the quality and the rigor in reporting were inconsistent.

Driven by concerns about the inconsistent characterization of MSC as well as different cell manufacturing protocols, the ISCT issued a statement article for minimal criteria for defining MSC in 2006.^5^ In this statement, the ISCT committee supported the use of the recommended designation “Multipotent mesenchymal stromal cells” for MSC^24^ and defined 3 minimal criteria to describe MSC: “(i) adherence to plastic; (ii) specific surface antigen expression; and (iii) multipotent differentiation potential.” This statement was updated in 2019, where the committee stressed again the importance of the cell nomenclature and the need for additional criteria to report such as tissue of origin and functional assays to better characterize these cells.^6^

Our results show that the uptake for the ISCT definition is inconsistent among a contemporary sample of articles. Most authors still use the term of “Mesenchymal Stem Cell” to describe MSC, even without providing any evidence of the stemness of their cells. Initial minimal criteria from the 2006 statement article, plastic adherence, cell markers, and differentiation assay were inconstantly reported 36%, 53%, and 40%, respectively, of our sample of studies. Most of the articles reporting the use of cell markers to describe their cell population did not provide any information on the flow cytometry cutoff used to define positive and negative markers. In a post hoc test, we examined the association between journal impact factor and level of completeness in reporting the minimal criteria to define MSC from the ISCT. We found no correlation between the level of completeness and the journal impact factor which is consistent with the systematic review from Saginur et al, where they described little to no association between journal impact factor and study methodological quality.^25^ In addition, RCT did not show a better completeness in reporting minimal criteria to define MSC compared to other clinical studies. Tissue source was the most reported ISCT criteria. The broad variety of tissue sources reported echoes with some authors’ concern that, considering cell markers are nonspecific and artifacts and misinterpretation are frequent in differentiation assays, so-called MSCs can be isolated from any kind of tissue.^26^ Another concern is that, in our study, only 20% of all articles (10% of the clinical studies) reported using a functional assay to describe MSCs’ potency and properties. These functional potency assays seem to be critical to better characterize MSC and provide a prediction of these MSCs’ effectiveness in clinical settings (depending on the disease, patient demographics),^4^ and are required for FDA biologics license application.^7^

We also found that critical information for MSC descriptions (eg, viability assessment and MSC fitness) and culture conditions (eg, oxygen level) were often missing. Among reported items, we found a wide variety of reported culture parameters such as number of passages and cell confluence in the different study categories. The importance of culture conditions in the field of MSC is well recognized as these conditions can dramatically change MSCs’ phenotype.^27^ MSCs may exhibit different functional properties depending on how they are produced, handled, and administered. In order to enhance reproducibility and transparency in MSC research, it is critical to report these important culture parameters as well as MSC viability and fitness.

In addition to these findings, we also showed in our sample of clinical studies using MSC an important variation in the MSC dose regimen (dose range was 10^5^ to 10^7^ cells/kg) and a number of MSC doses (range from 1 to 24 doses). Although MSC administration protocols (administration route, dose, number of doses) were overall well reported, other important information about the cell product used was absent. For instance, only 33% of the clinical articles reported a MSC viability assay at some point in their protocol and most of them did not provide the results of this assay. Administration of senescent or apoptotic cells can mitigate the results in terms of efficacy but also raise the concern about safety as these cells secrete or release paracrine factors which can negatively regulate the host cells. Some authors have reported that the acute inflammation triggered by the dead stem cells could be at the origin of tissue regeneration, more than the cell product itself. ^28^ Similarly, functional assays were reported in only 10% of the clinical studies. As stated above these functional assays provide insight into the MSC potency and their potential effectiveness in human diseases.

Overall, these findings from clinical studies are in line with a recent meta-analysis reported that MSC administration seems to be safe in humans but also stressed that the study design quality and reporting transparency of the included studies was sub-optimal.^10^ It is critical to have an extensive characterization of the MSC product used as well as a detailed cell manufacturing process to ensure reproducibility, comparison, and transparency between clinical trials using MSC. In addition, given indications that some trial participants have been “non-responders” to MSC therapy, unraveling this heterogeneous response to therapy and the development of predictive biomarkers will ultimately rely on the use of well-characterized and “standardized” MSC products.^4^

A key challenge of this scoping review was managing the large amount of preclinical and clinical MSC research being produced to create our evidence map. To address this challenge, we limited the literature search to 3 months, categorized the selected studies according to the research field (in vitro, animal, human), and for data charting, we randomly selected samples for each category with more than 100 included articles. A potential limitation of this scoping review was our choice to exclude non-English articles, meaning we may have missed relevant information published in another language.

## Conclusion

Our study highlighted a broad variability in reporting quality and completeness in both MSC definition and product characterization. This finding is of concern as many authors consider that for MSCs the “process is the product,” stressing the importance of limiting the sources of variability in the resulting cell product by clearly defining the cell (source, functional assays) and culture condition.^27^ In the light of the new evidence provided by our study, it is not surprising that there have been calls to “clearing-up the stem cells mess.”^3^ Therefore, we propose to develop a research protocol combining a rigorous consensus development method (modified Delphi method)^16^ to address the lack of consensus definition for MSC and to provide reporting guidelines for clinical studies using MSC.^15^ Equally important are strategies to support its dissemination and implementation. A science-based approach such as “integrated knowledge translation”^29^ may help by engaging knowledge users in the Delphi process since inception and developing a tailored end of project knowledge translation plan to support dissemination and implementation of the Delphi results. Results of this scoping review will be used to generate our initial Delphi survey and to identify potential participants among corresponding authors of the selected articles to contribute to the development of an internationally accepted consensus definition of MSCs.

## Supplementary Material

szab009_suppl_Supplementary_Figure_S1

szab009_suppl_Supplementary_Figure_S2

szab009_suppl_Supplementary_Table_S1

szab009_suppl_Supplementary_Table_S2

szab009_suppl_Supplementary_Table_S3

szab009_suppl_Supplementary_Table_S4

szab009_suppl_Supplementary_Table_S5

szab009_suppl_Supplementary_Material

szab009_suppl_Supplementary_Checklist

## Data Availability

No new data were generated or analyzed in support of this research.
