# Supplementary figures and images for: Definition and Characteristics of Mesenchymal Stromal Cells in Preclinical and Clinical Studies: A Scoping Review
Source: Stem Cells Transl Med. 2022 Feb 23;11(1):44–54. doi: 10.1093/stcltm/szab009 (PMC8895491; doi:10.1093/stcltm/szab009)

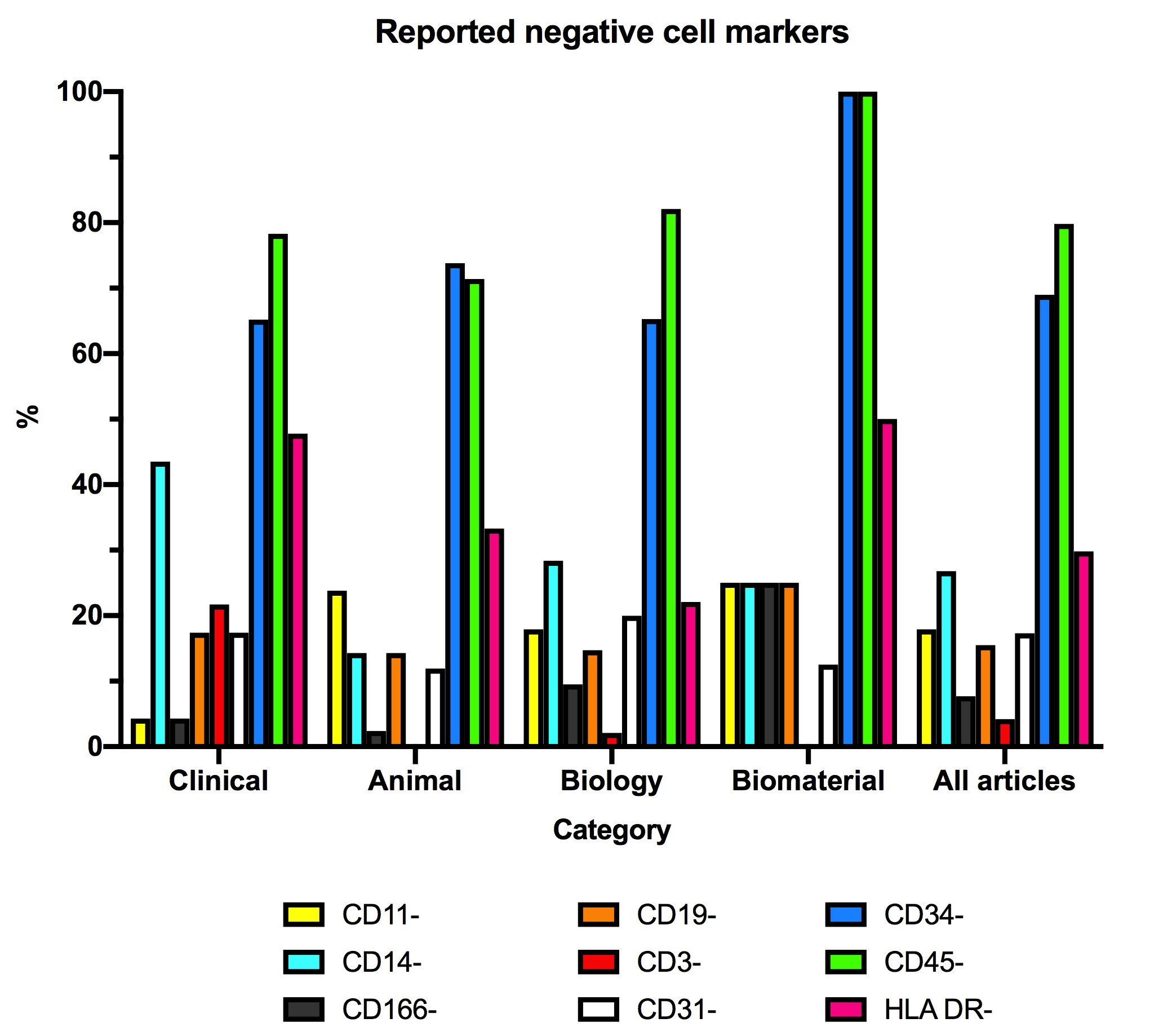

Supplement: szab009_suppl_Supplementary_Figure_S1 [file szab009_suppl_supplementary_figure_s1.jpeg]

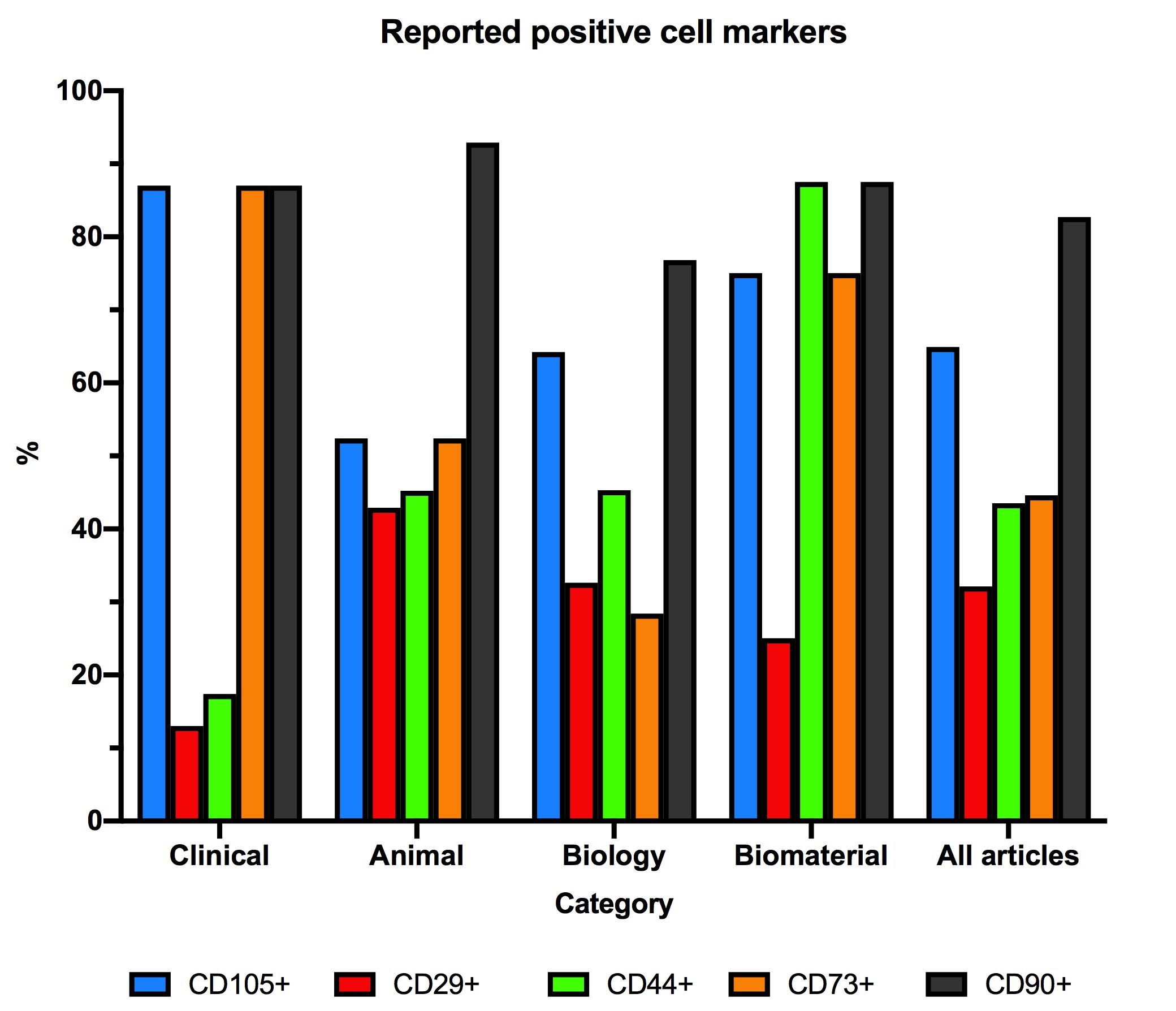

Supplement: szab009_suppl_Supplementary_Figure_S2 [file szab009_suppl_supplementary_figure_s2.jpeg]
