# Supplementary material for: Definition and Characteristics of Mesenchymal Stromal Cells in Preclinical and Clinical Studies: A Scoping Review
Source: Stem Cells Transl Med. 2022 Feb 23;11(1):44–54. doi: 10.1093/stcltm/szab009 (PMC8895491; doi:10.1093/stcltm/szab009)
Supplement: szab009_suppl_Supplementary_Table_S1 [file szab009_suppl_supplementary_table_s1.docx]

**Supplemental Material. Table S1 Article epidemiological characteristics: Journals, countries and funding sources.**

NGO: Non-governmental Organization

| **Journal** | **Country** | **Funding reported?** | **Funding sources** |
| --- | --- | --- | --- |
| **Clinical studies (n=42)** | | | |
| Stem Cell Research & Therapy | China | Yes | Reported as not funded |
| Stem Cell Reviews and Reports | Iran | Yes | Academic |
| Critical Care Medicine | Taiwan | Yes | Academic, Hospital |
| International Journal of Clinical and Experimental Medicine | China | No | - |
| Journal of International Medical Research | China | Yes | Foundation / charity / NGO, Hospital |
| Case reports in dermatological medicine | United States | No | - |
| JBRA Assisted Reproduction | Argentina | No | - |
| Stem cells international | Russia | Yes | Hospital |
| Archives of orthopaedic and trauma surgery | Korea | No | - |
| Molecular therapy | Spain | Yes | Academic, Foundation / charity / NGO, Hospital |
| Ebio Medicine | Spain | Yes | Government, Academic, Foundation / charity / NGO, Industry |
| The journal of sexual medicine | United States | Yes | Reported as not funded |
| Cytotherapy | Ireland | Yes | Government, Academic, Hospital |
| JBJS case connect | India | No | - |
| Inflammatory bowel diseases | United States | Yes | Foundation / charity / NGO, Hospital |
| Journal of musculoskeletal and neuronal interactions | China | Yes | Government |
| Aging and disease | China | Yes | Government, Academic |
| Stem cell research & therapy | Vietnam | Yes | Government |
| Knee surgery, sports traumatology, arthroscopy | Greece | Yes | Reported as not funded |
| Cells | Poland | Yes | Government |
| Knee surgery, sports traumatology, arthroscopy | Korea | Yes | Reported as not funded |
| Bratislava medical journal | Turkey | No | - |
| Arthroscopy: the journal of arthroscopic and related surgery | Korea | Yes | Government |
| Journal of clinical medicine | Poland | Yes | Government |
| Translational stroke research | France | Yes | Government |
| Regenerative Therapy | Japan | Yes | Reported as not funded |
| Stem Cell Research & Therapy | Sweden | Yes | Government, Foundation / charity / NGO, Hospital |
| International Orthopaedics | France | No | - |
| Stem Cells Translational Medicine | United States | No | - |
| Injury | Spain | Yes | Government |
| Journal of Immunology Research | Iran | Yes | Academic |
| Investigative Ophthalmology & Visual Science | Spain | Yes | Government, Foundation / charity / NGO, Industry |
| American Journal of Transplantation | Netherlands | No | - |
| Stem cells and development | Germany | Yes | Foundation / charity / NGO |
| Neural Regeneration research | China | Yes | Government, Hospital |
| Engineering | China | Yes | Government |
| Plastic and Reconstructive Surgery | Brazil | No | - |
| Stem Cells Translational Medicine | Italy | Yes | Foundation / charity / NGO |
| Journal of Crohn's and Colitis | Netherlands | Yes | Foundation / charity / NGO |
| Kidney International | United States | Yes | Government, Hospital |
| Cell Transplantation | Taiwan | Yes | Reported as not funded |
| Neurology | United States | Yes | Industry |
| **Animal studies (n=77)** | | | |
| International journal of stem cells | Egypt | No | - |
| Cellular immunology | China | Yes | Government, Foundation / charity / NGO |
| FASEB journal | China | Yes | Government, Academic, Hospital |
| Neurobiology of disease | China | Yes | Government |
| Stem cell research & therapy | China | Yes | Government |
| Journal of cellular and molecular medicine | China | Yes | Government, Academic, Hospital |
| Rejuvenation research | China | Yes | Government, Academic, Hospital |
| Cellular immunology | China | Yes | Government |
| Molecular neurobiology | Argentina | Yes | Government |
| Stem cells international | China | Yes | Government, Hospital |
| International journal of medical sciences | China | Yes | Government |
| Translational psychiatry | Korea | Yes | Government |
| ACS biomaterials science & engineering | China | Yes | Government, Hospital |
| Molecular medicine reports | China | Yes | Government |
| The American journal of sports medicine | Singapore | Yes | Government |
| Stem cells | China | Yes | Academic, Hospital |
| Stem cells international | United States | Yes | Academic |
| Cell biochemistry & function | China | Yes | Government, Hospital |
| Stem cells international | China | Yes | Government |
| Frontiers in cellular neuroscience | China | Yes | Government, Hospital |
| Tissue and cell | China | Yes | Government |
| International journal of physiology, pathophysiology and pharmacology | China | No | - |
| Journal of biomedical materials research | United States | Yes | Government, Academic |
| Journal of diabetes investigation | Japan | Yes | Industry |
| Theranostics | China | Yes | Government |
| Journal of biomedical materials research | China | Yes | Government |
| Polymers | Taiwan | Yes | Government, Hospital |
| Journal of global pharma technology | Egypt | Yes | Government |
| Plos one | United States | Yes | Foundation / charity / NGO, Industry |
| European review for medical and pharmacological sciences | China | Yes | Government |
| Translational stroke research | United Kingdom | Yes | Government, Academic |
| Stem cells | Korea | No | - |
| Stem cells and development | Canada | Yes | Government, Academic |
| Plastic and reconstructive surgery global open | France | Yes | Industry |
| Hand | United States | Yes | Reported as not funded |
| Materials science & engineering C | India | Yes | Academic |
| The anatomical record | United States | No | - |
| Stem cells and development | Brazil | Yes | Government, Academic |
| Experimental and therapeutic medicine | China | Yes | Government |
| Stem cell research & therapy | China | Yes | Government |
| Gynecologic and obstetric investigation | China | Yes | Foundation / charity / NGO |
| Oxidative medicine and cellular longevity | China | Yes | Government, Academic |
| Diabetes Research and Clinical Practice | China | Yes | Government, Foundation / charity / NGO |
| International Journal of Stem Cells | China | Yes | Government |
| Molecular Therapy | China | Yes | Government, Academic |
| International Journal of Stem Cells | China | Yes | Government |
| International journal of neuroscience | China | Yes | Government |
| Journal of Musculoskeletal and Neuronal Interactions | China | Yes | Unclear |
| Cell Transplantation | China | Yes | Government, Foundation / charity / NGO |
| Journal of Experimental & Clinical Cancer Research | Italy | Yes | Government |
| Respirology | United States | Yes | Government, Academic |
| Critical Care Medicine | United States | Yes | Government |
| Journal of Neurochemistry | United States | Yes | Government, Academic |
| Journal of biomedical materials research | Iran | Yes | Academic |
| Stem Cell Research & Therapy | China | Yes | Government |
| Journal of Biochemical and Molecular Toxicology | China | Yes | Government |
| Stem Cell Research & Therapy | Japan | Yes | Academic, Foundation / charity / NGO |
| Journal of Controlled Release | Korea | Yes | Government |
| Biomaterials | United States | Yes | Government, Academic |
| Urology Journal | China | No | - |
| Stem cells and development | China | Yes | Government |
| Frontiers in Immunology | Czechia | Yes | Government, Academic |
| Aging Cell | China | Yes | Government, Academic |
| Regenerative medicine | India | Yes | Academic |
| Journal of Controlled Release | China | Yes | Government, Academic |
| Blood Advances | United States | Yes | Government, Academic |
| Biomolecules | Iran | Yes | Academic, Foundation / charity / NGO |
| Journal of Diabetes Research | China | Yes | Government |
| Frontiers in Neurology | Mexico | Yes | Government, Academic |
| American Journal of Transplantation | India | Yes | Government |
| Biomedical Materials | India | No | - |
| Medicina | Saudi Arabia | Yes | Academic |
| Aging | China | Yes | Unclear |
| Macedonian Journal of Medical Sciences | Saudi Arabia | Yes | Reported as not funded |
| Neurosurgery | United States | Yes | Academic, Foundation / charity / NGO |
| Transplantation | Italy | Yes | Government, Foundation / charity / NGO |
| Journal of Materials Chemistry B | Taiwan | No | - |
| **Biology studies (n=160)** | | | |
| Future science OA | Portugal | Yes | Hospital |
| International journal of molecular sciences | Germany | Yes | Reported as not funded |
| Biotechnology & bioengineering | United States | Yes | Industry |
| Veterinary research communications | Brazil | Yes | Government, Foundation / charity / NGO |
| Stem cell research & therapy | United States | Yes | Government |
| Cell journal | Iran | Yes | Academic |
| Stem cells | Netherlands | Yes | Government |
| Biochemical and biophysical research communications | China | Yes | Government |
| Orthopaedic surgery | China | Yes | Government |
| Oxidative medicine and cellular longevity | China | Yes | Government |
| Journal of biochemistry | China | Yes | Government |
| ACS biomaterials sconce & engineering | Korea | Yes | Government, Foundation / charity / NGO |
| Frontiers in physiology | China | Yes | Government, Academic, Foundation / charity / NGO |
| Journal of cellular and molecular medicine | China | Yes | Government, Academic |
| Journal of ginseng research | Korea | Yes | Government |
| FASEB journal | China | Yes | Government |
| Materials | Japan | Yes | Foundation / charity / NGO |
| Biomedical research international | China | Yes | Government, Academic |
| Acta histochemica | China | Yes | Reported as not funded |
| Cytotherapy | China | Yes | Government |
| Leukemia | United States | Yes | Government, Academic, Foundation / charity / NGO, Industry |
| Stem cells and development | United States | No | - |
| Journal of biomedical materials research | Germany | Yes | Hospital |
| Cell death & disease | Korea | Yes | Government, Hospital |
| Modern rheumatology | China | Yes | Government  Academic |
| Acta histochemica | China | Yes | Government |
| Orthopaedic surgery | China | Yes | Government |
| Journal of biomedical materials research | China | Yes | Academic, Hospital |
| Nanoscale | China | Yes | Academic |
| Molecular medicine reports | China | Yes | Government |
| Journal of cellular and molecular medicine | China | Yes | Government, Academic, Foundation / charity / NGO |
| Molecular medicine reports | China | Yes | Government, Academic |
| Biotechnology letters | China | Yes | Government |
| Stem cell research & therapy | Spain | Yes | Government, Academic |
| Strahlentherapie und onkologie | Germany | Yes | Unclear |
| Theranostics | Germany | Yes | Government, Academic |
| Liver transplantation | United States | Yes | Foundation / charity / NGO, Hospital |
| Biology open | China | Yes | Government, Academic |
| Medical science monitor | China | No | - |
| Indian journal of clinical biochemistry | Iran | Yes | Government |
| Stem cell reviews and reports | United States | Yes | Government, Academic |
| Glycoconjugate journal | United States | Yes | Government, Academic |
| Stem cells international | Korea | Yes | Government |
| Toxicology in vitro | Korea | Yes | Government |
| Cells | Korea | Yes | Government, Industry |
| Journal of tissue engineering | Germany | Yes | Government, Academic |
| BioMedical research international | United States | Yes | Academic |
| Science advances | France | Yes | Unclear |
| Tissue engineering | United States | Yes | Academic, Foundation / charity / NGO |
| Biomolecules | Spain | Yes | Government |
| Journal of cellular and molecular medicine | Iran | Yes | Academic |
| Experimental cell research | Germany | Yes | Reported as not funded |
| Vascular pharmacology | United States | Yes | Academic |
| Cells | Greece | Yes | Government |
| Cell proliferation | China | Yes | Government, Academic |
| Cancer gene therapy | Spain | Yes | Government, Foundation / charity / NGO |
| Medical mycology | Colombia | Yes | Academic |
| Molecular & cellular proteomics | Spain | Yes | Government |
| Pharmaceutics | Italy | Yes | Government |
| Annals of vascular surgery | Italy | No | - |
| Asian pacific journal of cancer prevention | Iran | Yes | Academic |
| International journal of clinical and experimental medicine | China | No | - |
| Biomedical research international | Korea | Yes | Government, Academic |
| Biomaterials | Netherlands | Yes | Foundation / charity / NGO |
| Frontiers in bioengineering and biotechnology | Netherlands | Yes | Government |
| Regenerative therapy | Japan | Yes | Government, Industry |
| Cells | Germany | Yes | Government, Foundation / charity / NGO |
| Methods | United States | Yes | Government |
| Journal of clinical biochemistry and nutrition | Japan | Yes | Government |
| Stem cells international | Poland | Yes | Government |
| Nanomaterials | Germany | Yes | Reported as not funded |
| Orthopaedic surgery | China | No | - |
| Scandinavian journal of immunology | China | Yes | Government |
| Journal of orthopaedic research | Netherlands | Yes | Unclear |
| Iranian biomedical journal | Iran | No | - |
| Journal of surgical research | United States | Yes | Government, Academic |
| World journal of stem cells | Greece | No | - |
| Cytotechnology | Egypt | Yes | Government |
| Cells tissues organs | France | Yes | Government, Academic |
| Journal of medical virology | China | Yes | Government, Academic |
| Journal of orthopaedic translation | China | Yes | Government |
| Biotechnology letters | China | Yes | Government, Academic |
| Journal of cellular physiology | China | Yes | Government, Academic |
| Stem cells international | China | No | - |
| Cell cycle | China | Yes | Government |
| Molecular medicine reports | China | Yes | Government |
| Immunology Letters | China | Yes | Government, Foundation / charity / NGO |
| Annals of Clinical & Laboratory Science | China | Yes | Academic |
| Journal of Cellular Physiology | China | Yes | Government, Foundation / charity / NGO |
| Acta Dermato-Venereologica | China | Yes | Government |
| Stem Cells and Development | China | Yes | Government |
| Bone Research | China | Yes | Government, Academic |
| Stem Cell Research | Korea | Yes | Government |
| Bioscience Reports | Korea | Yes | Government |
| BioTechniques | China | Yes | Government, Foundation / charity / NGO |
| Journal of Orthopaedic Surgery and Research | Korea | Yes | Government |
| European Journal of Pharmacology | Russia | Yes | Government |
| Cells | Korea | Yes | Government |
| Leukemia | United States | Yes | Government, Foundation / charity / NGO |
| European Review for Medical and Pharmacological Sciences | China | No | - |
| Journal of Biomedical Material Research | China | Yes | Government |
| Cell & Bioscience | China | Yes | Government, Foundation / charity / NGO |
| Journal of Cellular and Molecular Medicine | China | Yes | Government, Hospital |
| Molecular Medicine REPORTS | China | Yes | Government, Foundation / charity / NGO |
| Journal of Tissue Engineering and Regenerative Medicine | United States | Yes | Foundation / charity / NGO |
| Cells | United Kingdom | Yes | Academic, Hospital |
| Tissue Engineering and Regenerative Medicine | Korea | Yes | Government |
| Biochemical and Biophysical Research Communications | China | Yes | Government |
| Biomedical Material | India | Yes | Government |
| Journal of Plastic, Reconstructive & Aesthetic Surgery | Germany | Yes | Government, Academic |
| Nanomedicine | China | Yes | Government, Academic |
| Molecular and Cellular Probes | China | Yes | Government |
| Cell Communication and Signaling | Canada | Yes | Academic, Foundation / charity / NGO |
| International journal of molecular medicine | China | Yes | Government |
| European Review for Medical and Pharmacological Sciences | China | Yes | Government, Academic |
| Stem Cell Research & Therapy | India | Yes | Government |
| Cellular and Molecular Biology | China | No | - |
| Oxidative Medicine and Cellular Longevity | Poland | Yes | Government |
| Tissue Engineering | United States | Yes | Government, Academic, Foundation / charity / NGO |
| Pharmacognosy Jounal | Indonesia | Yes | Reported as not funded |
| International journal of molecular sciences | Italy | Yes | Hospital |
| Cancer Letters | Switzerland | Yes | Government, Foundation / charity / NGO |
| PLOS ONE | Germany | Yes | Academic |
| Journal of Stomatology, Oral and Maxillofacial Surgery | Germany | Yes | Foundation / charity / NGO |
| Lasers in Medical Science | Iran | Yes | Academic |
| In vitro Cellular & Developmental Biology-animal | Egypt | No | - |
| Stem Cell Research & Therapy | China | Yes | Government |
| Journal of Tissue Engineering and Regenerative Medicine | Italy | Yes | Government, Foundation / charity / NGO |
| Colloids and Surfaces B: Biointerfaces | Portugal | Yes | Government  Foundation / charity / NGO |
| Journal of clinical medicine | United Kingdom | Yes | Government |
| Cell cycle | China | No | - |
| Cell Biology International | China | Yes | Government |
| Cell Proliferation | China | Yes | Government, Academic |
| Neuroscience Letters | China | Yes | Government |
| International Journal of Medical Sciences | Korea | Yes | Government, Academic |
| Stem Cell Research & Therapy | Denmark | Yes | Government, Foundation / charity / NGO |
| Molecular Biology Reports | Russia | Yes | Government |
| Journal of Biomedical Optics | Russia | Yes | Government |
| British Journal of Haematology | Italy | Yes | Foundation / charity / NGO |
| Stem Cell Research & Therapy | Germany | Yes | Academic |
| FEBS Open Bio | Germany | Yes | Government, Academic |
| Injury | Greece | No | - |
| Stem Cells | Portugal | Yes | Government, Foundation / charity / NGO |
| Acta Biomaterialia | Japan | Yes | Industry |
| Biomédica | Venezuela | Yes | Government, Academic |
| J Biomed Mater Res | India | Yes | Government |
| Scandinavian Journal of Clinical and Laboratory Investigation | Sweden | No | - |
| Molecules | Saudi Arabia | Yes | Academic |
| Science Advances | United States | Yes | Government |
| Journal of Food Biochemistery | India | Yes | Academic |
| American Journal of Physiology- Gastrointestinal and Liver Physiology | United States | Yes | Government  Academic |
| Bioelectricity | United States | Yes | Government, Foundation / charity / NGO |
| International journal of molecular and cellular medicine | Egypt | No | - |
| Journal of International Medical Research | China | Yes | Government |
| Brazilian Journal of Pharmaceutical Sciences | China | Yes | Government |
| The FASEB Journal | United States | Yes | Government, Foundation / charity / NGO |
| Cytometry Part A | China | Yes | Government, Academic |
| BMC Research Notes | Iran | Yes | Academic |
| Bio Techniques | Switzerland | Yes | Foundation / charity / NGO |
| eLife | Denmark | Yes | Government, Academic, Foundation / charity / NGO |
| **Biomaterial studies (n=39)** | | | |
| ACS applied materials & interfaces | Netherlands | Yes | Academic |
| Biomaterials | United States | Yes | Government, Academic |
| Journal of biomedical materials research | China | Yes | Government |
| Journal of biomaterials applications | China | Yes | Academic |
| Macromolecular bioscience | Belgium | Yes | Government, Foundation / charity / NGO |
| Clinical hemorheology and microcirculation | Germany | Yes | Academic |
| Journal of materials chemistry B | China | Yes | Government, Academic |
| Materials | Spain | Yes | Academic, Foundation / charity / NGO |
| Bioimpacts | Iran | Yes | Government |
| International journal of nanomedicine | United States | Yes | Government |
| Journal of biomedical materials research | Poland | Yes | Government |
| Stem cells | United States | Yes | Government, Academic |
| ACS biomaterials science & engineering | United States | Yes | Government, Academic, Industry |
| Acta biochimica et biophysica sinica | China | Yes | Government |
| ACS applied materials & interfaces | Taiwan | Yes | Government, Academic |
| Journal of tissue engineering and regenerative medicine | Korea | Yes | Government |
| Stem cells international | France | Yes | Government, Academic, Foundation / charity / NGO |
| Small | China | Yes | Government, Academic |
| International journal of biological macromolecules | Iran | No | - |
| Materials science & engineering C | Austria | Yes | Academic |
| ACS biomaterials science & engineering | China | Yes | Government, Academic |
| Advanced healthcare materials | Italy | No | - |
| Materials science & engineering C | Iran | Yes | Government, Foundation / charity / NGO |
| Journal of Biomedical Material Research | Taiwan | Yes | Hospital |
| Biomaterials | United Kingdom | Yes | Academic, Foundation / charity / NGO, Industry |
| ACS Appl. Mater. Interfaces | Canada | Yes | Government, Academic |
| Biomedical Material | Pakistan | Yes | Academic |
| Journal of Materials Chemistry B | China | Yes | Government |
| ACS Biomaterial Science & Engineering | United States | Yes | Government, Academic |
| Biomedical Materials | China | Yes | Government |
| Journal of Materials Chemistry B | China | Yes | Government |
| Journal of Materials Chemistry B | United States | Yes | Government, Academic, Foundation / charity / NGO |
| Journal of Materials Chemistry B | China | Yes | Government |
| Journal of Materials Chemistry B | China | Yes | Government, Academic |
| Journal of Materials Chemistry B | China | Yes | Government, Academic |
| Journal of Materials Chemistry B | China | Yes | Government |
| Journal of Materials Chemistry B | Singapore | Yes | Academic |
| Journal of Materials Chemistry B | Portugal | Yes | Government |
| Journal of Materials Chemistry B | Spain | Yes | Government |
