# Supplementary material for: Definition and Characteristics of Mesenchymal Stromal Cells in Preclinical and Clinical Studies: A Scoping Review
Source: Stem Cells Transl Med. 2022 Feb 23;11(1):44–54. doi: 10.1093/stcltm/szab009 (PMC8895491; doi:10.1093/stcltm/szab009)
Supplement: szab009_suppl_Supplementary_Table_S2 [file szab009_suppl_supplementary_table_s2.docx]

**Supplemental Table S2. Disease models used in clinical and animal studies.**

1. **Clinical studies.**

| Disease category | n | % | Disease (n) |
| --- | --- | --- | --- |
| Cancer and blood disorders | 2 | 5 | Advanced solid tumor (1), graft versus host disease (1) |
| Cardiovascular system | 2 | 5 | Myocardial infarction (1), critical limb ischemia (1) |
| Digestive system | 3 | 7 | Crohn (2), liver cirrhosis (1) |
| Endocrine system | 0 | 0 | - |
| Eye, ear, throat, periodontology | 1 | 2 | Keratoconus (1) |
| Genitourinary system | 6 | 14 | Kidney transplant (2), infertility (2), erectile dysfunction (1), atherosclerotic renovascular disease (1) |
| Musculoskeletal system | 12 | 29 | Knee osteoarthritis (6), Duchenne muscular dystrophy (1), rotator cuff disease (1), long bone non-unions (1), Avascular Necrosis (1), bone defect post osteomyelitis (1), Refractory Rheumatoid Arthritis (1) |
| Nervous System | 6 | 14 | Spinal cord injury (1), minimally conscious state (1), ischemic stroke (1), cerebral adrenoleukodystrophy (1), acute complete spinal cord injury (1), amyotrophic lateral sclerosis (1) |
| Respiratory system  and critical care medicine | 7 | 17 | COPD (2), vocal fold scarring, ARDS (2), COVID-19 pneumonia (2) |
| Skin and subcutaneous tissue | 3 | 7 | Psoriasis (1), chronic non healing wounds (1), photoaging (1) |

1. **Animal studies.**

| Disease category | n | % | Disease model (1) |
| --- | --- | --- | --- |
| Cancer and blood disorders | 9 | 12 | Graft-versus-Host disease (4), acute radiation sickness (2), bone marrow damage of aging (1), Hepatocellular carcinoma (1), osteosarcoma (1) |
| Cardiovascular system | 9 | 12 | Ischemic heart injury (7), Right ventricle hypertrophy (1), myocardial fibrosis diabetic (1) |
| Digestive system | 4 | 5 | Chronic pancreatitis (1), liver fibrosis (1), toxic colitis (1), post-surgery intra-peritoneal adhesions (1) |
| Endocrine system | 2 | 3 | Type 2 diabetes mellitus (2) |
| Eye, ear, throat, periodontology | 5 | 6 | Diabetic retinopathy (1), palate cleft (1), cochlear toxicity (1), maxillary alveolar bone defect (1), Dental implant in a Diabetic model (1), |
| Genitourinary system | 8 | 10 | Acute kidney injury (1), renal fibrosis (1), kidney transplantation (1), intra-uterine adhesion (1), diabetic nephropathy (4) |
| Musculoskeletal system | 12 | 16 | Bone defect (5), osteochondral defects (3), osteoarthritis (2), diabetic myopathy (1), flexor tendon injuries (1) |
| Nervous System | 18 | 23 | Degenerative diseases (Alzheimer 2, Parkinson 1, Huntington 1, Diabetes related cognitive impairment 1), spinal cord injury (3), stroke (ischemic 1, hemorrhagic 2), pain (deafferentation pain 1, sciatic 1), intervertebral discs annular injury (1), autoimmune encephalomyelitis (1), schizophrenia (1), temporal lobe epilepsy (1), traumatic brain injury (1) |
| Respiratory system and critical care medicine | 6 | 8 | Bronchopulmonary dysplasia (2), Sepsis (2), pulmonary fibrosis (1), phosphagen induced ALI (1) |
| Skin and subcutaneous tissue | 4 | 5 | Wound healing (2), burns (1), atopic dermatitis (1) |
