# Supplementary material for: Definition and Characteristics of Mesenchymal Stromal Cells in Preclinical and Clinical Studies: A Scoping Review
Source: Stem Cells Transl Med. 2022 Feb 23;11(1):44–54. doi: 10.1093/stcltm/szab009 (PMC8895491; doi:10.1093/stcltm/szab009)
Supplement: szab009_suppl_Supplementary_Table_S3 [file szab009_suppl_supplementary_table_s3.docx]

**Supplemental table S3. Report of the intervention group in Animal studies.**

|  | | | n (%) |
| --- | --- | --- | --- |
| Administration route reported | | | **77 (100)** |
|  | Intra-venous* | | 33 (43) |
|  | Intra-tracheal | | 4 (5) |
|  | Intra-muscular | | 0 (0) |
|  | Intra-thecal | | 1 (1) |
|  | Intra-articular | | 1 (1) |
|  | **Other routes** | | 40 (52) |
|  |  | Intra-tumor (cancer) | 1 (3) |
|  |  | Cardiovascular (intra-myocardium) | 8 (20) |
|  |  | Digestive system | 2 (5) |
|  |  | Ear-nose-throat | 2 (5) |
|  |  | Eye | 1 (3) |
|  |  | Genitourinary system | 1 (3) |
|  |  | Musculoskeletal system (intra-tendon) | 10 (25) |
|  |  | Nervous System | 5 (13) |
|  |  | Skin and subcutaneous tissue | 10 (25) |
| MSC dose reported | | | **70 (91)** |
|  | Dose cells/kg | | 17 (24) |
|  |  | Range 1.25x10^5^ to 10^7^ cells/kg | |
|  | Total cells | | 50 (71) |
|  |  | Range 3x10^4^ to 10^7^ cells per dose | |
|  | Other dose report | | 3 (4) |
|  |  | Dose reported in cell/surface scaffold or media to incubate suture | |
|  | Use different doses | | 5 (7) |
|  | Single administration | | 59 (84) |
|  | Multiple administration | | 11 (16) |
|  |  | Range 2 to 6 doses | |

*2 studies used 2 administration routes (IV and other)
