# Supplementary material for: Definition and Characteristics of Mesenchymal Stromal Cells in Preclinical and Clinical Studies: A Scoping Review
Source: Stem Cells Transl Med. 2022 Feb 23;11(1):44–54. doi: 10.1093/stcltm/szab009 (PMC8895491; doi:10.1093/stcltm/szab009)
Supplement: szab009_suppl_Supplementary_Table_S4 [file szab009_suppl_supplementary_table_s4.docx]

**Supplemental Table 4. Comparison of journals impact factors and completeness of report for minimal criteria to define MSC (ISCT 2006).**

|  |  |  | **Reported minimal criteria ISCT 2006** | | | |
| --- | --- | --- | --- | --- | --- | --- |
|  |  |  | **None** | **1** | **2** | **3** |
| Clinical studies (n=42) | n | | 15 | 16 | 8 | 3 |
|  | % | | 36 | 38 | 19 | 7 |
|  | Journal Impact factor | Median | 4.8 | 5.4 | 5.7 | 6.1 |
|  |  | Min | 1.3 | 3.1 | 4.1 | 5.4 |
|  |  | Max | 11.4 | 10.6 | 6.9 | 6.8 |
|  |  | N/A | 2 | 1 | 1 | 1 |
| Animal studies (n=77) | n | | 28 | 10 | 29 | 10 |
|  | % | | 36 | 13 | 38 | 13 |
|  | Journal Impact factor | Median | 4.9 | 5.3 | 5.4 | 4.0 |
|  |  | Min | 2.0 | 3.3 | 1.5 | 2.5 |
|  |  | Max | 9.8 | 11.4 | 12.5 | 11. 6 |
|  |  | N/A | 1 | 1 | 2 | - |
| Biology studies (n=160) | n | | 36 | 45 | 40 | 39 |
|  | % | | 23 | 28 | 25 | 24 |
|  | Journal Impact factor | Median | 3.8 | 3.6 | 4.4 | 4.0 |
|  |  | Min | 1.3 | 1.5 | 1.3 | 0.3 |
|  |  | Max | 11.6 | 11.5 | 14.1 | 13.6 |
|  |  | N/A | 2 | 1 | 3 | 1 |
| Biomaterial studies (n=39) | n | | 22 | 8 | 6 | 3 |
|  | % | | 56 | 21 | 15 | 8 |
|  | Journal Impact factor | Median | 6.3 | 5.6 | 5.1 | 6.3 |
|  |  | Min | 2.4 | 2.6 | 3.6 | 3.8 |
|  |  | Max | 13.3 | 12.5 | 7.3 | 12.5 |
|  |  | N/A | - | - | - | - |

The three minimal criteria to define MSC from the initial ISCT publication (Dominici et al. 2006) include plastic adherence, cell markers and *in vitro* differentiation assays.
