# Supplementary material for: Definition and Characteristics of Mesenchymal Stromal Cells in Preclinical and Clinical Studies: A Scoping Review
Source: Stem Cells Transl Med. 2022 Feb 23;11(1):44–54. doi: 10.1093/stcltm/szab009 (PMC8895491; doi:10.1093/stcltm/szab009)
Supplement: szab009_suppl_Supplementary_Table_S5 [file szab009_suppl_supplementary_table_s5.docx]

**Supplemental table 5. Comparison between randomized controlled trials (RCT) and other clinical studies.**

|  | **Reported minimal criteria ISCT 2006** | | | | **Most reported cell markers** |
| --- | --- | --- | --- | --- | --- |
|  | None | 1 | 2 | 3 |  |
| **RCT**  (n=9) | 3 | 3 | 2 | 1 | - CD73+ (n=2) - CD90+ (n=2) - CD105+ (n=2) - CD44+ (n=2) - CD45- (n=2) |
| **Other**  (n=33) | 12 | 12 | 7 | 2 | - CD73+ (n=18) - CD90+ (n=18) - CD105+ (n=18) - CD45- (n=16) - CD34- (n=14) |

Minimal criteria to define MSC from the initial ISCT publication (Dominici et al. 2006) include plastic adherence, cell markers and *in vitro* differentiation assays.
