# Supplementary material for: Definition and Characteristics of Mesenchymal Stromal Cells in Preclinical and Clinical Studies: A Scoping Review
Source: Stem Cells Transl Med. 2022 Feb 23;11(1):44–54. doi: 10.1093/stcltm/szab009 (PMC8895491; doi:10.1093/stcltm/szab009)
Supplement: szab009_suppl_Supplementary_Material [file szab009_suppl_supplementary_material.docx]

**Supplemental material. Search Strategy.**

Database: Embase Classic+Embase <1947 to 2020 June 17>, Ovid MEDLINE(R) ALL <1946 to June 16, 2020>

Search Strategy:

--------------------------------------------------------------------------------

1 exp Mesenchymal Stem Cells/ (95944)

2 exp Mesenchymal Stem Cell Transplantation/ (22989)

3 exp Multipotent Stem Cells/ (45073)

4 exp Mesenchymal Stromal Cells/ (50042)

5 ((mesenchymal adj3 (stem or stroma$1 or progenitor*)) and cell$1).tw. (127324)

6 (mesenchymal adj2 (stem or stromal or progenitor or multipotent or bone marrow or adipose or placenta*)).tw,kw. (128850)

7 (MSC or MSCs or ADMSC or ADMSCs or BM-MSC or BM-MSCs or BMD-MSC or BMD-MSCs or BMDMSC or BMDMSCs).tw. (80734)

8 ((multipotent or multi-potent) adj3 (stroma$1 cell$1 or stem cell$1)).tw. (11104)

9 marrow stroma$1 cell$1.tw. (17065)

10 (colony-forming unit fibroblast* or CFU-F$1).tw. (2271)

11 Mesoderm/cy (5790)

12 1 or 2 or 3 or 4 or 5 or 6 or 7 or 8 or 9 or 10 or 11 (187059)

13 limit 12 to english language (175240)

14 limit 13 to abstracts (168800)

15 (202003* or 202004* or 202005*).dt. (357770)

16 14 and 15 (1795)

**17 16 use medall (1795) Medline**

18 *mesenchymal stem cell/ (57226)

19 *mesenchymal stem cell transplantation/ (16103)

20 *mesenchymal stroma cell/ (6557)

21 (mesenchymal adj5 (cell* or stem or stromal or progenitor or multipotent or bone marrow or adipose or placenta*)).tw. (184136)

22 (((multipotent or multi-potent) adj3 (stem or stroma$1 or progenitor*)) and cell*).tw. (15646)

23 (marrow stroma* adj2 cell*).tw. (17877)

24 18 or 19 or 20 or 21 or 22 or 23 (213240)

25 limit 24 to english language (200006)

26 limit 25 to abstracts (196523)

27 conference abstract.pt. (3813150)

28 26 not 27 (167626)

29 (202003* or 202004* or 202005*).dc. (570221)

30 28 and 29 (2758)

**31 30 use emczd (2758) Embase**

32 17 or 31 (4553)

33 remove duplicates from 32 (3339)

**34 33 use medall (1784) Medline**

**35 33 use emczd (1555) Embase**
